# Nut Cracked? Does the ACORN Trial End the Debate Surrounding Vancomycin and Piperacillin-Tazobactam Combination Therapy and Increased Risk for Acute Kidney Injury?

**DOI:** 10.1093/ofid/ofad645

**Published:** 2023-12-18

**Authors:** Jason M Pogue, Samuel L Aitken

**Affiliations:** Department of Clinical Pharmacy, College of Pharmacy, University of Michigan, Ann Arbor, Michigan, USA; Department of Clinical Pharmacy, College of Pharmacy, University of Michigan, Ann Arbor, Michigan, USA; Department of Pharmacy Services, Michigan Medicine, Ann Arbor, Michigan, USA

**Keywords:** ACORN, acute kidney injury, nephrotoxicity, piperacillin-tazobactam, vancomycin

## Abstract

Observational data published over the past decade have suggested that concomitant receipt of piperacillin-tazobactam with vancomycin significantly increases the risk for vancomycin-associated acute kidney injury. Importantly, however, there is significant controversy surrounding this association, and debate continues about the veracity of the risk. Given this ongoing debate, the recently published “Cefepime vs Piperacillin-Tazobactam in Adults Hospitalized With Acute Infection: The ACORN Randomized Clinical Trial” is of tremendous interest to the infectious diseases community. In ACORN, the authors conclude that there was no association between receipt of cefepime or piperacillin-tazobactam and the primary outcome of acute kidney injury or death by day 14, despite the fact that >75% of the population received concomitant vancomycin. In this perspective, we provide a brief history on the controversy, provide a critical analysis of the ACORN findings, and ultimately discuss how these data help inform the ongoing debate.

Vancomycin is the most widely used antibiotic in US hospitals [1], and data suggest that vancomycin-associated acute kidney injury (VA-AKI) occurs in up to 43% of treated patients [2]. While antimicrobial stewardship programs have implemented strategies over the past decade to decrease the incidence of VA-AKI, with some notable successes including dose optimization and limiting durations of therapy, rates are still unacceptably high, and further strategies to limit this devastating adverse event are urgently needed.

Over the past decade, numerous retrospective analyses have identified concomitant administration of vancomycin with piperacillin-tazobactam (VPT) as a putative novel risk factor for VA-AKI [3–9]. A recent systematic review and meta-analysis concluded that use of the VPT combination was associated with significantly increased risk of VA-AKI in adults (adjusted odds ratio, 3.15; 95% CI, 1.72–5.76) and children (odds ratio, 4.55; 95% CI, 2.71–10.21) [10]. However, the reported association between VPT and VA-AKI has been surrounded by significant controversy for a number of reasons. First, the association has not been universal, and multiple studies have failed to demonstrate any increased VA-AKI risk associated with VPT [11–13]. Furthermore, the association between VPT and VA-AKI lacks a mechanistic understanding and has not been demonstrated in animal models [14]. Finally, given the retrospective and observational nature of published studies reporting increased VA-AKI risk with VPT, study design limitations, such as residual confounding by indication, may explain the reported association. Conversely, the association between vancomycin-flucloxacillin combination therapy and increased risk of kidney injury that was demonstrated in the CAMERA-2 randomized clinical trial [15] has been recapitulated in animal models [16] similar to those that failed to show an association between VPT and VA-AKI.

Notably, the association between VPT and VA-AKI is further confounded by the use of serum creatinine (Scr) as the gold standard for measuring kidney function and diagnosing AKI. Although Scr is largely filtered by the glomerulus, proximal tubular secretion accounts for approximately 10% to 20% of creatinine elimination in patients with normal renal function and an even higher percentage in patients with renal insufficiency [17, 18]. Substances that inhibit steps in these secretory pathways can cause an elevation in Scr, even in the absence of damage to the kidney, [19] and evidence exists that piperacillin may inhibit these pathways [20]. Thus, in patients receiving VPT, increases in Scr might not be indicative of actual kidney injury. Indeed, in patients receiving VPT, limited observational data suggest a possible disconnect between elevations in Scr and serum cystatin C, a potentially unbiased marker of kidney function [21]. The lack of clarity regarding the veracity of the association between VPT and VA-AKI has left clinicians and antimicrobial stewards unclear on the optimal management strategy for patients requiring broad-spectrum therapy.

It is in this backdrop that the ACORN randomized clinical trial [22] comparing the safety of empiric therapy with cefepime vs piperacillin-tazobactam in patients with suspected infection aimed to enhance our muddled understanding of this clinical controversy. ACORN was a revolutionary trial in that the investigators randomized eligible patients at the prescriber level, within the electronic medical record, with an informed consent waiver from the institutional review board given the equipoise to the clinical question at hand. Using this methodology, the investigators were impressively able to enroll >2500 patients with suspected infection into the primary analysis population at 1 site in <1 year, with an average time to enrollment of 1.2 hours from presentation. The primary outcome in ACORN was the highest stage of AKI (defined by increases in Scr) or death between randomization and day 14, measured on a 5-level ordinal scale (0, no death or AKI; 1–3, stage 1–3 AKI; 4, death).

The investigators demonstrated no significant difference in the incidence of acute kidney injury or death by day 14 among patients empirically receiving cefepime or piperacillin-tazobactam (odds ratio, 0.95; 95% CI, .80–1.13). As >75% of patients in each arm were receiving vancomycin at baseline and >80% received at least 1 dose in the first 14 days, these results on the surface suggest that VPT does not appreciably increase the risk of AKI.

However, to interpret how these data inform the debate surrounding vancomycin and piperacillin-tazobactam as a cause of AKI, a deeper dive is necessary. First, note that this randomized controlled trial was performed in patients with suspected infection. That is, this question targets empiric therapy with cefepime or piperacillin-tazobactam in patients who may—or may not—need these drugs. This empiric usage is reflected in an average duration of therapy of 3 days (IQR, 1–4) with the study drug (ie, cefepime or piperacillin-tazobactam) and an average duration of vancomycin of 2 days (IQR, 1–4). Additionally, although most patients were receiving vancomycin at the time of enrollment, the degree of overlap between the administration times of vancomycin and the study drugs is not reported, and the durations of therapy listed here are described as the total durations of therapy over the first 14 days of the study period. Furthermore, only 54% of patients presented with sepsis, and the percentage of the cohort who ultimately had positive cultures at any point during admission was lower still at 29%. Unfortunately, there is no mention made with respect to (1) the percentage of patients in the cohort who ultimately were proven to have an infection (even if no positive microbiology), (2) the appropriateness of empiric therapy, (3) the in vitro activity of study drug, or (4) the time to appropriate therapy in patients who were infected. These points are critical because each variable affects the association between the study arm and death, which is important, as the AKI outcomes incorporate death into the hierarchical endpoints regardless of the relevance of treatment assignment to death. For example, if a patient is ultimately not infected, what role would cefepime or piperacillin-tazobactam be expected to play in one’s death? The answer to this question is likely “none.” However, if deaths are similar in study arms in patients who were uninfected, this does bias the primary outcome toward the null, given that any trends in stage of AKI between patients receiving cefepime and piperacillin-tazobactam would be muted by no difference at the top of the hierarchy (ie, a score of 4 for death). Unless the investigators suggest that there may be an efficacy difference between the empiric antibiotics or there is a significant concern that the antibiotic is going to cause an adverse event leading to death by day 14, the death component should have been removed from the primary endpoint. At the very least, a strong case could be made for censoring deaths, rather than placing them as the worst value of an ordinal outcome.

The concern with the role that death may play on the outcomes in ACORN becomes exacerbated with a closer look at the baseline characteristics of the 2 study arms. When compared with patients who were randomized to piperacillin-tazobactam, those randomized to receive cefepime were more likely at baseline to be in the intensive care unit (6.5% vs 4.2%), to be receiving mechanical ventilation (9.1% vs 7.3%), and to be at stage ≥2 AKI (29.6% vs 25.8%). Furthermore, patients randomized to cefepime were more likely to have baseline coma (6.9% vs 5.9%) or delirium (5.1% vs 3.9%). These differences in baseline characteristics suggest that patients randomized to receive cefepime were at somewhat increased risk for death (as well as acute kidney injury) over the assessment period. Indeed, Supplementary Figure 4 suggests a significant increase in death or receipt of new kidney replacement therapy in patients receiving cefepime on day 1 of the study drug, whereas Supplementary Figures 5 and 6 suggest similar increases on day 1 of stage ≥2 AKI as well as coma/delirium in said patients. Consistent with the slight baseline differences between the arms, the rate of death by day 14 was numerically higher (7.6% vs 6.0%, *P* = .13) in patients receiving cefepime, which affected the primary outcome of highest AKI stage or death by day 14. As it relates to AKI, the known lag time between damage to the nephron and elevations in Scr further supports that these differences on day 1 are unrelated to the study drug [23, 24]. For VA-AKI, the reported median onset of AKI is between 4 and 17 days [2]; thus, the duration of therapy of the study drugs—and vancomycin, if one assumes that administration was for the same duration as the study drugs—may be too short to truly test the association.

With these 2 things in mind (ie, imbalances in study arms and expected onset of VA-AKI), some interesting data are presented in the supplementary appendix of the article to suggest that there may in fact be an increased risk of AKI in ACORN in patients receiving vancomycin in combination with piperacillin-tazobactam. Table 1 is adapted from Supplementary Table 11 and presents comparative AKI rates with increasing durations of the study drug (ie, cefepime or piperacillin-tazobactam) and, for previously mentioned reasons, excludes patients who died from the assessment. While there is a numerical association between receipt of piperacillin-tazobactam and AKI across all analyses, the incidence becomes statistically higher (via chi-square test) in patients who receive therapy lasting ≥72 hours (28.5% vs 22.9%, *P* = .03) and ≥96 hours (34.3% vs 26.8%, *P* = .02). This is consistent with a close inspection of Supplementary Figure 5, which demonstrates that the increases in stage ≥2 AKI in patients randomized to cefepime on day 1 reverse over time, with the end probability of stage ≥2 AKI being higher in patients receiving piperacillin-tazobactam. If one performs a similar analysis, again excluding deaths and further limiting the cohort to those who received vancomycin (also in Supplementary Table 11), the incidence of AKI is also statistically higher in patients receiving piperacillin-tazobactam (232/924 [25.1%] vs 173/858 [20.2%], *P* = .01). Of note, these analyses are not perfect by any stretch of the imagination. For one, it is unclear if patients who died experienced AKI prior to death. If there were patients who had AKI prior to death, excluding them from an AKI assessment would be inappropriate. Second, these are subgroup and sometimes post hoc analyses where we are not adjusting for multiplicity, and these differences in key subgroups may be due to chance or residual imbalances between groups once randomization has been broken. Furthermore, as mentioned in the introduction, AKI in this evaluation is defined by rises in Scr, which may be subject to a pseudotoxicity due to the inhibition of tubular secretion of creatinine by piperacillin. That said, a closer look at these data demonstrates that the bulk of the difference in rates of AKI between patients receiving cefepime and piperacillin-tazobactam is in stage 2 or 3 AKI, which is a higher degree of creatinine elevation than that expected from secretion inhibition alone [19] and therefore suggests true kidney injury.

**Table 1.** Comparative Rates of AKI as a Function of Duration of Therapy of Cefepime vs Piperacillin-Tazobactam, Excluding Patients Who Died in ACORN

	Any Duration		≥48 h		≥72 h		≥96 h	
AKI: Stage	CEF (n = 1122)	PT (n = 1219)	CEF (n = 774)	PT (n = 901)	CEF (n = 597)	PT (n = 713)	CEF (n = 392)	PT (n = 464)
Any	212 (18.9)	267 (21.9)	166 (21.4)	222 (24.6)	137 (22.9)	203 (28.5)*	105 (26.8)	159 (34.3)*
1^a^	86 (7.7)	100 (8.2)	62 (8.0)	77 (8.5)	48 (8.0)	71 (10.0)	36 (9.2)	49 (10.6)
2^b^	41 (3.7)	70 (5.7)	33 (4.2)	62 (6.9)	29 (4.9)	60 (8.4)	22 (5.6)	48 (10.3)
3^c^	85 (7.6)	97 (8.0)	71 (9.2)	83 (9.2)	60 (10.0)	72 (10.1)	47 (12.0)	62 (13.4)

Abbreviations: AKI, acute kidney injury; FEP, cefepime; TZP, piperacillin-tazobactam.

Data are listed as No. (%).

^a^Stage 1: an elevation in creatinine level that was 1.5–1.9 times the baseline level or increased by ≥0.3 mg/dL.

^b^Stage 2: an elevation in creatinine level that was 2.0–2.9 times the baseline value.

^c^Stage 3: an elevation ≥3.0 times the baseline value.

^*^ *P* < .05 for TZP vs FEP in this subgroup.

So, what does it all mean? Perhaps most important, despite the issues raised in this commentary, the truly innovative methodology in this trial is a Herculean accomplishment, and we can only hope that it sets the precedent for future trials in infectious diseases. It also likely demonstrates that in the empiric setting, when combined with good stewardship to limit the duration of therapy to ∼48 hours, piperacillin-tazobactam and cefepime are similarly safe from a kidney injury perspective, even if most patients get some vancomycin sprinkled in. Thus, other factors can drive this empiric treatment decision (eg, local susceptibility, microbiological and antibiotic histories, and spectrum of activity considerations). This finding is consistent with data in the intensive care unit setting demonstrating no association with short-course VPT and an increased risk of AKI [11]. Unfortunately, ACORN does not meaningfully add to the conversation of whether combination therapy with vancomycin and piperacillin-tazobactam beyond 48 hours increases the risk of vancomycin-associated AKI. With limitations noted and fully acknowledged, the analyses provided in this commentary suggest that even within ACORN there are hints of increased risks of AKI in patients receiving piperacillin-tazobactam, particularly as the duration of therapy increases. This finding is consistent with published literature on the topic. Yet, the lack of clarity in receipt of concomitant vancomycin and the use of Scr to measure renal function are major limitations in any such interpretation. Even if all patients in the analyses presented herein were on concomitant vancomycin, elevations in Scr are not a new finding in patients receiving this combination, and these data cannot address whether true AKI or simply pseudo-AKI was being assessed. Thus, further study—in patients receiving longer durations of combination therapy and with use of markers other than creatinine—is needed to definitively ascertain the safety of the combination.

## Notes

***Patient consent statement***. As this is a commentary on a clinical controversy, it does not include factors necessitating patient consent.
